# Selpercatinib and capmatinib combination promotes sustained complete response in novel *ISOC1-RET* fusion lung cancer after resistance to *RET* inhibitor via *MET* amplification: Case Report

**DOI:** 10.3389/fonc.2023.1264231

**Published:** 2023-10-09

**Authors:** Caio Abner Leite, Raíssa Pierri Carvalho, Felipe Marques da Costa, Augusto Kreling Medeiros, Fabio Augusto Schutz, William Nassib William

**Affiliations:** ^1^ Department of Clinical Oncology, Hospital Beneficência Portuguesa de São Paulo, São Paulo, Brazil; ^2^ Oncoclinicas, São Paulo, Brazil

**Keywords:** lung adenocarcinoma, *RET* fusion, *MET* amplification, selpercatinib, capmatinib

## Abstract

*RET* fusions occur in 1–2% of non-small cell lung cancer. Selpercatinib and pralsetinib are selective *RET* inhibitors with significant improvement of outcome in patients with tumor harboring *RET* fusion; however, resistance mechanisms appear frequently, mainly driven by MAPK pathway bypass, secondary *RET* mutations, or in 5% via *MET* amplification. Co-inhibition of *RET* and *MET* is a compelling strategy for overcoming *MET*-dependent resistance to *RET* inhibitors and potentially other inhibitors. To our knowledge, this is the first report of a novel *ISOC1-RET* fusion lung cancer with a durable complete response to selpercatinib, with resistance via *MET* amplification, which was overcome by the successful combination of selpercatinib and capmatinib.

## 1 Introduction

The *RET* proto-oncogene encodes a transmembrane tyrosine kinase part of the embryological development of the nervous, gastrointestinal, and genitourinary systems. *RET* can be abnormally activated by rearrangements or mutations. *RET* gene fusions or rearrangements occur in 1–2% of non-small cell lung cancer (NSCLC), of which 70-90% have kinesin family 5B (*KIF5B*) and 10-25% have coiled-coil domain containing 6 (*CCDC6*) as fusion partner (1).

Selpercatinib is a selective *RET* inhibitor with significant antitumor activity in patients with lung cancer and *RET* fusion (objective response rate of 64%), including those who previously received at least one platinum-based chemotherapy, multikinase inhibitors, and/or immunotherapy as shown in a phase I/II study (2). Nonetheless, the vast majority of patients who respond to selpercatinib will eventually progress, illustrating the need to characterize and develop strategies to overcome resistance mechanism. Acquired selpercatinib resistance has been shown to be driven by signaling that bypasses the MAPK pathway, secondary *RET* mutations, or in 5% via *MET* amplification (3).

The co-inhibition of *RET* and *MET* was used as a strategy to overcome *MET*-dependent resistance to *RET* inhibitors. Clinical benefit was seen in one patient who received crizotinib (*MET/ALK/ROS1* inhibitor) added to selpercatinib (3) another patient when added to pralsetinib (4) and stable disease in one patient who received selpercatinib combined with capmatinib (*MET* selective inhibitor) (5). Here, we report the first case of a patient with NSCLC harboring *ISOC1-RET* fusion with a sustained complete response to selpercatinib that progressed after acquiring *MET* amplification, but responded completely to the combination of selpercatinib and capmatinib.

## 2 Case report

A 57-year-old female, non-smoker, diagnosed with stage IIIA NSCLC in 2012, was treated with lower left lobectomy and adjuvant cisplatin plus pemetrexed for four cycles.

In 2019, she presented with multiple small metastatic lung nodules. An attempt at a CT-guided biopsy in the lung failed to acquire significant amounts of tumor DNA for molecular analysis (FoundationOne CDx, Foundation Medicine, Cambridge, MA). The initial tumor from 2012 was sent for molecular analysis and did not show driver gene alterations, but the sample was classified as unqualified (FoundationOne CDx, Foundation Medicine, Cambridge, MA). Therefore, she was treated with pembrolizumab, carboplatin, and pemetrexed for 4 cycles, followed by pembrolizumab and pemetrexed q3 weeks for 6 months, with stable disease as the best response.

**Figure 1:**
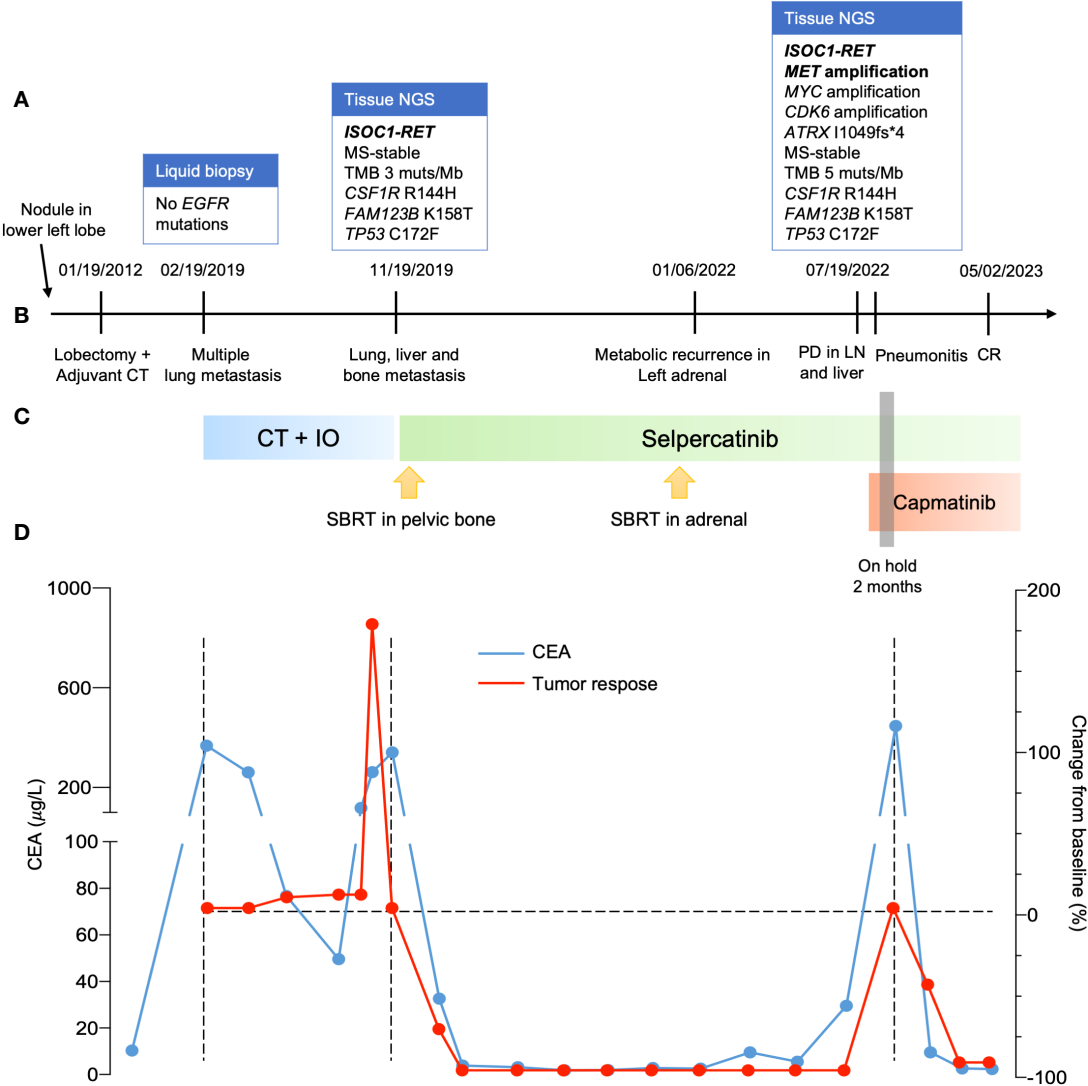
Clinical progress of the patient. **(A)** Molecular profiling when progressed to metastatic disease, after the first line and when failure to selpercatinib. **(B)** timeline of clinical progress. **(C)** systemic treatment used. **(D)** curves with evolution of CEA in blue during patient treatment and change of tumor size evaluated by RECIST version 1.1. NGS, next-generation sequence; CT, computed tomography; PD, progressive disease; LN, lymph node; CR, complete response; CT, chemotherapy; IO, immuno-oncology; CEA, carcinoembryonic antigen; RECIST, response evaluation criteria in solid tumors.

**Figure 2:**
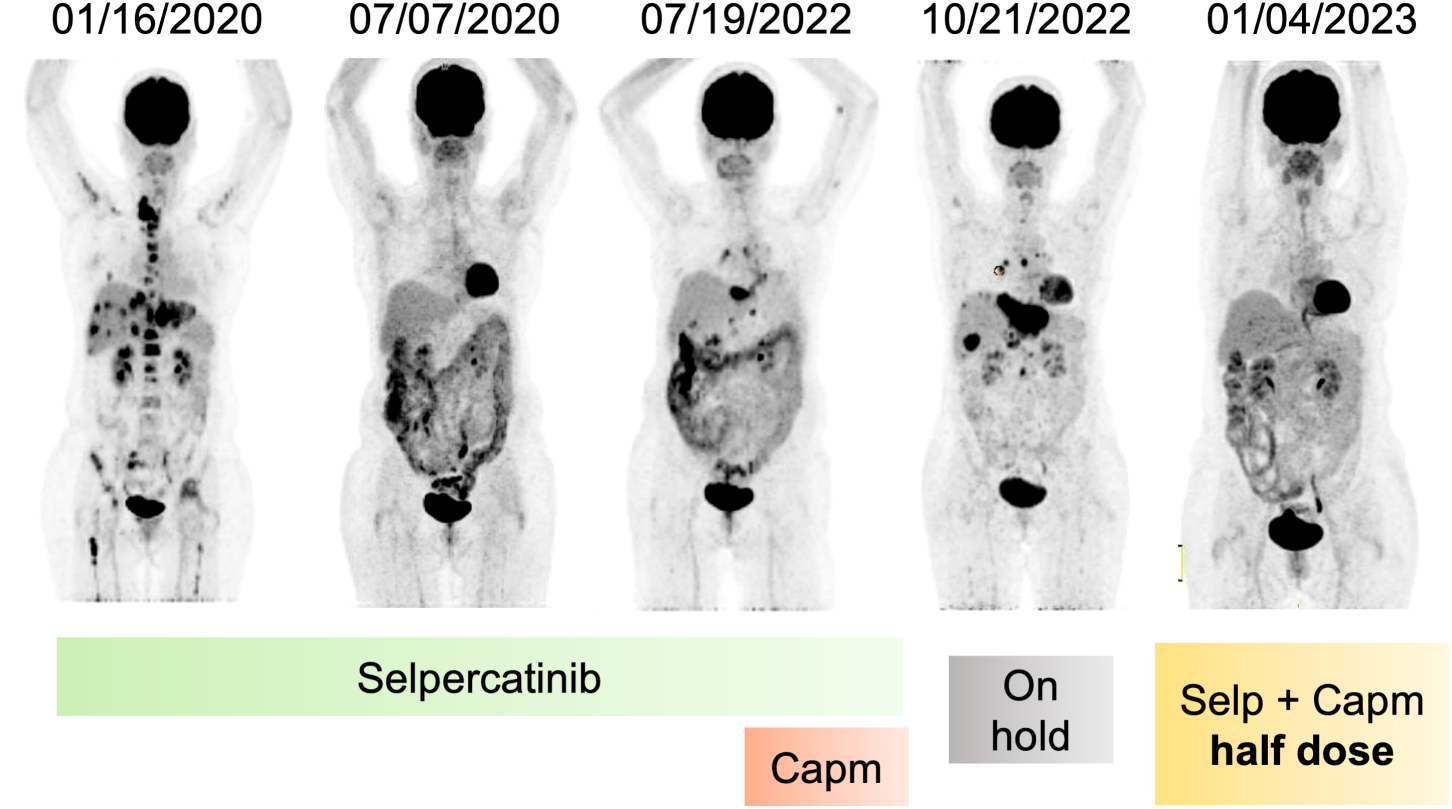
Metabolic response of patient after RET inhibition and resistance. Coronal PET images evidencing metabolic resolution by 30 months during use of selpercatinib, followed by recurrence and association with capmatinib, stopping treatment due to adverse effects, and again metabolic resolution after reintroduction of selpercatinib and capmatinib in half dose. Capm, capmatinib; Selp, selpercatinib; PET, positron emission tomography.

After progression to first-line therapy in December 2019, a new biopsy (neck lymph node) was performed and sent for molecular analysis. Tumor NGS (FoundationOne CDx, Foundation Medicine, Cambridge, MA) identified an *ISOC1-RET* fusion (breakpoint in intron 1 of *ISOC1* gene, chr5:128440428-128441063 and intron 11 of *RET*, chr10:43611181-43611710). She started treatment with selpercatinib 160 mg q12h on a compassionate use program (**Figure 1**). She experienced a steep drop in her CEA levels within the first 3 months of treatment (**Figure 1**) and a complete response on imaging (**Figures 1** and **2**), accompanied by weight recovery and improved bone pain and fatigue. During treatment with selpercatinib, the patient developed grade 2 diarrhea and bilateral leg edema, which were managed with dietary changes, antidiarrheals and diuretics. 24 months after selpercatinib was started, the patient developed metabolic activity in the left adrenal gland, which was treated with stereotactic body radiotherapy.

**Figure 3:**
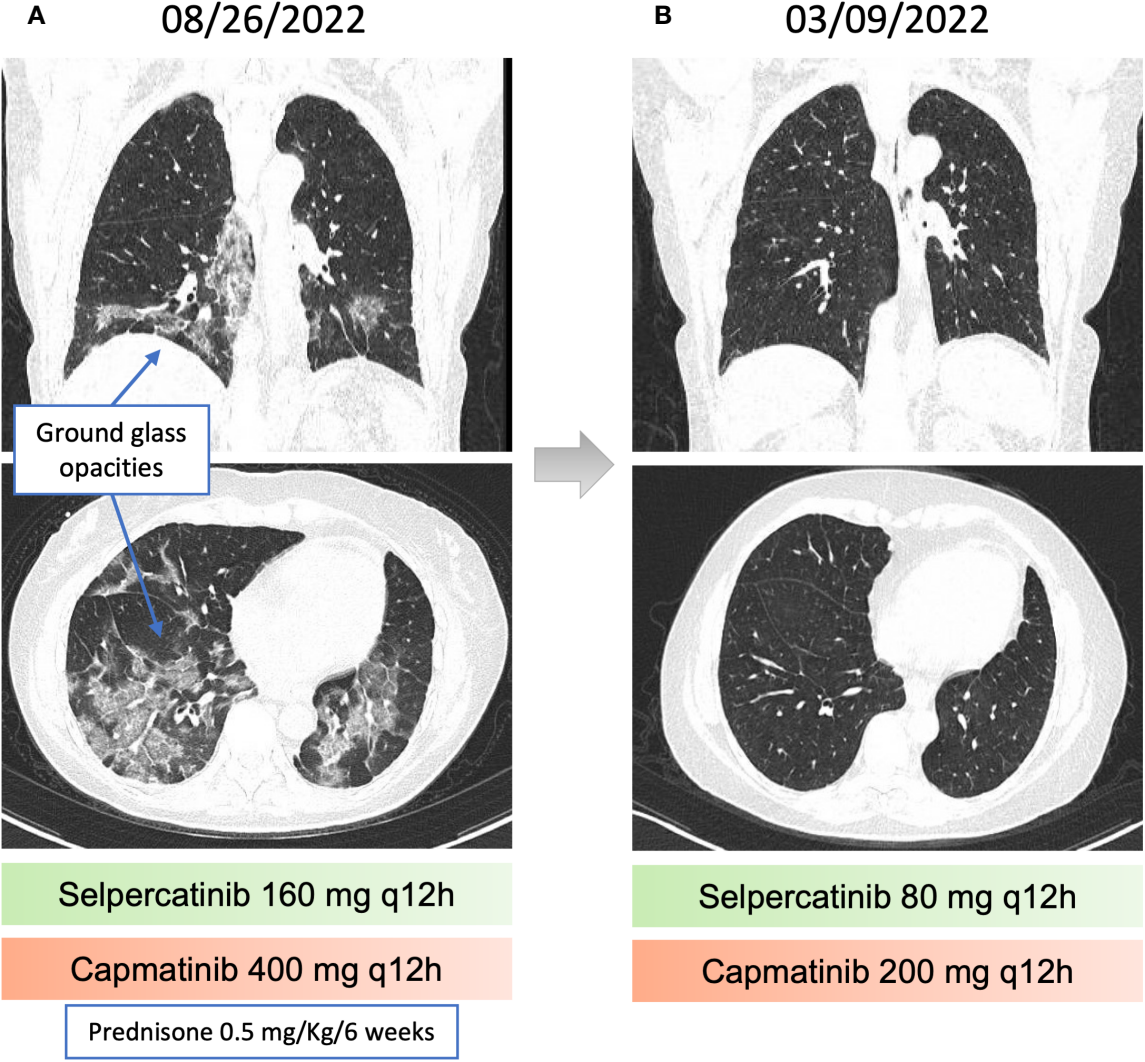
Pneumonitis related to capmatinib and recovery after dose reduction. **(A)** chest computed tomography (CT) revealing Ground-glass opacities, associated with perilobular consolidation components, distributed in peribronchovascular and peripheral regions, suggestive of organizing pneumonia. **(B)** Resolution of the findings described in the previous chest CT.

The disease progressed in multiple lymph nodes, the left adrenal gland and the liver, and rising CEA levels after 30 months of selpercatinib use, when *ISOC1-RET* fusion remained and *MET* amplification of 29 copies was identified as a resistance mechanism by tissue NGS from a biopsy of the left adrenal gland (**Figure 1**). She then initiated treatment with capmatinib 400 mg q12h and maintained selpercartinib, and within 1 week the CEA levels dropped. However, she developed grade 3 pneumonitis (dyspnea, hypoxemia, and ground-glass opacities on chest computed tomography, as depicted in **Figure 3**) with no infectious or autoimmune disease, and both drugs were held. After steroid use, the patient had a quick recovery. She remained off therapy for 2 months. During that time, she presented symptoms consistent with progressive disease in the liver, with abdominal pain, as well as rising CEA levels (**Figure 1**). She was re-started on capmatinib and selpercatinib both at half-dose. After 3 months, the patient experienced a complete response by RECIST version 1.1, with normalization of CEA levels (**Figures 1** and **2**) and no disease-related symptoms or medication-related adverse events. This response holds up to now, for at least 8 months.

## 3 Discussion

In this report, we demonstrate that (1) the novel *ISOC1-RET* fusion induces *RET*-dependent tumor growth that can be impaired by *RET* inhibition with selpercatinib (2); *MET* amplification is a mechanism of resistance to selpercatinib in this context (3); the use of the specific *MET* inhibitor capmatinib with selpercatinib may overcome resistance; but (4) this combination may trigger pneumonitis. Lastly (5), rechallenge with selpercatinib and capmatinib both at half dose restored tumor control without pneumonitis recurrence. The features of this case are clinically relevant and may inform clinical practice as more patients are expected to develop resistance to *RET* inhibitors with increasing use of these drugs worldwide.

This is the first report of *ISOC1* (intron 1)- *RET* (intron (11)) reciprocal fusion. Therefore, the *ISCO1-RET* fusion protein in this case retained the tyrosine kinase domain of *RET*, conferring a potential oncogenic activity. Consistently with our case, the most frequent breakpoint of *RET* occurs in intron 11 as demonstrated in a study with 9101 patients with NSCLC, of which 167 had *RET* rearrangement. This study demonstrated also that there was no significant difference in survival between different breakpoints of *RET (*intron 11 *vs*. other locations) (6).

Isochorismatase domain-containing protein 1 (*ISOC1*) is a hydrolase that catalyzes the transformation of isochorismate into 2,3-dihydroxy-2,3-dihydrobenzoate and pyruvate. The human *ISOC1* gene is located on chromosome 5q23.3 and encodes a protein of 298 amino acids that is physiologically related to eye, testis, and neutrophil development. Additionally, overexpression of *ISOC1* could increase the proliferation, viability, migration, and invasion of cancer cells, leading to a significantly worse disease-free survival in NSCLC patients (7). Data from the phase I/II study using pralsetinib, another *RET* inhibitor, suggest that patients with *CCDC6-RET* may have a better prognosis than those with *KIF5B-RET* (progression-free survival not reached versus 12,8 months), but data are scarce for other *RET* partners (8).

In the first line, our patient had a PFS of 9 months, which is very similar to the 8.8 months found in the KEYNOTE-189 study (9). Therefore, *RET* gene fusion did not interfere with the outcome to first-line treatment. Consonantly, a retrospective study of NSCLC patients, which were stratified according to *RET* status, demonstrated that there were no significant differences in survival by *RET* status among patients with lung adenocarcinoma treated with standard first-line therapy (10). In second line, selpercatinib provided our patient with a 30 month PFS, which was longer than the median of LIBRETTO study (22 months), reaching sustained complete response with good quality of life (2).

When our patient progressed to selpercatinib, *MET* amplification was found as an acquired off-target resistance mechanism to selpercatinib in line with what has been previously described (10). Although this is known mechanism of resistance, data on efficacy and toxicity combined *MET* and *RET* inhibition in this context are scarce (3–5).

Capmatinib is a *MET* inhibitor approved to treat NSCLC patients with *MET* exon 14 skipping mutations. Capmatinib has also been shown to be active in the setting of *MET* amplification (albeit not approved for this specific indication), especially with gene copy number of 10 or higher, when overall response was observed in 29% of patients (11, 12). Specifically, the combination of selpercatinib and capmatinib was first reported in a patient with *KIF5B-RET* NSCLC that developed a *MET* amplification of 12 copies. Although no severe toxicities were seen in that case, the best response was, disappointingly, stable disease for 4.5 months only (5). As MET amplification is a known resistance mechanism to treatments with inhibitors of other TKIs, the addition of capmatinib to treatment with EGFR and ALK inhibitors has been studied with promising efficacy and acceptable tolerance (13, 14).

Selpercatinib causes diarrhea, dry mouth, and fatigue (2). Capmatinib can cause peripheral edema, nausea, and vomiting as the main adverse events. Dyspnea secondary to capmatinib has been reported (7% grade 3 or 4), as well as one death due to pneumonitis (12). In our case, a few days of full dose combination was sufficient to induce pneumonitis requiring hospitalization and temporary treatment interruption. Rechallenge with the same combination at half dose was successful, with sustained complete response after 7 months of therapy, and peripheral edema and diarrhea as the main and manageable adverse effects.

## 4 Conclusions

This is the first report of *ISOC1-RET* fusion in a patient with NSCLC treated with selpercatinib, with a durable complete response, but subsequent acquired resistance secondary to *MET* amplification. Although we were able to overcome this *MET*-dependent resistance with a combination of selpercatinib and capmatinib, severe drug-induced pneumonitis emerged. This illustrates the need for prospective trials assessing safety of this combination, as well as dose optimization, since our patient’s disease was controlled with lower doses of the drugs. These data are not only immediately relevant for clinicians considering off label use of these drugs, but may also inform development of strategies to treat resistance of NSCLCs driven by other RTK, as *MET* amplification has commonly been described in those settings.

## Data availability statement

The raw data supporting the conclusions of this article will be made available by the authors, without undue reservation.

## Ethics statement

Ethical review and approval was not required for the study on human participants in accordance with the local legislation and institutional requirements. Written informed consent was obtained from the participant/patient(s) for the publication of this case report.

## Author contributions

CL: Writing – original draft, Writing – review & editing. RC: Writing – review & editing. FC: Writing – review & editing. AM: Writing – review & editing. FS: Writing – review & editing. WW: Writing – review & editing.
